# Paediatric low-grade glioma: the role of classical pathology in integrated diagnostic practice

**DOI:** 10.1007/s00381-024-06591-6

**Published:** 2024-09-18

**Authors:** Thomas J. Stone, Ashirwad Merve, Fernanda Valerio, Shireena A. Yasin, Thomas S. Jacques

**Affiliations:** 1 Developmental Biology and Cancer Research and Teaching Department, UCL GOS Institute of Child Health, London, UK; 2 Department of Histopathology, Great Ormond Street Hospital, London, UK; 3 Division of Neuropathology, The National Hospital for Neurology and Neurosurgery, London, UK

**Keywords:** Glioma, Nervous system neoplasms, MAP kinase, Neuropathology

## Abstract

Low-grade gliomas are a cause of severe and often life-long disability in children. Pathology plays a key role in their management by establishing the diagnosis, excluding malignant alternatives, predicting outcomes and identifying targetable genetic alterations. Molecular diagnosis has reshaped the terrain of pathology, raising the question of what part traditional histology plays. In this review, we consider the classification and pathological diagnosis of low-grade gliomas and glioneuronal tumours in children by traditional histopathology enhanced by the opportunities afforded by access to comprehensive genetic and epigenetic characterisation.

## Introduction

Low-grade gliomas are the most common central nervous system (CNS) tumours in children and contribute to a vast burden of morbidity. Pathological diagnosis has become a critical part of their clinical care, partly due to the increasing complexity of the tumour types as molecularly novel entities are defined but also due to the increased role of targeted treatments that rely on a fully integrated molecular diagnosis.

In this review, we focus on those tumours that are low-grade (defined as CNS WHO grade 1 or 2), show glial or glioneuronal differentiation and frequently arise in childhood. We describe the ‘classical’ histopathology, i.e. the histological features. However, while morphology remains the foundation of diagnosis, molecular findings are critical to a firm diagnosis, and we will discuss the tests that ensure a reliable diagnosis for clinical treatment.

### Clinical importance of pathological diagnosis

Low-grade gliomas have a good overall survival in most cases, and only particular subtypes (e.g. pleomorphic xanthoastrocytoma) carry a realistic risk of transformation to a higher grade. Nonetheless, the impact on patients in terms of long-term disability cannot be underestimated (see e.g. [1]), and patients are often committed to long-term therapy and follow-up. Therefore, it is probably better to consider them as chronic diseases than typical neoplasms [2].

In this context, it is worth considering the role of pathological diagnosis, particularly when historically, some subsets of low-grade glioma are reliably diagnosed without a tissue diagnosis. The most important role of pathology is to confirm that the tumour is a low-grade glioma and exclude higher-grade differential diagnoses. Moreover, pathological sub-typing offers the best chance of guiding conventional adjuvant therapy and offers prognostic reassurance to the patient and family.

The shift in clinical treatment towards the routine use of first-line targeted therapy necessitates tissue diagnosis, and this is reflected not only in routine practice but also in recommendations for trial design [3]. Furthermore, the long-term use of targeted therapies raises the question of how the biology of these tumours changes over a long time, particularly following treatment. Such tissue changes are likely to determine the length of the treatment and the likelihood of rebound on withdrawal of treatment. Therefore, a better understanding of the pathology of long-term disease is likely to be of great importance in the future.

### Approach to diagnosis

It is against this diverse set of goals for tissue diagnosis that a discussion of the most helpful classification and diagnostic tools can be set. In conversations that focus on the classification of paediatric gliomas, a degree of controversy arises around the most appropriate modality to classify and diagnose the tumours. Views vary as to the relative value of histology, sequencing-based techniques and methylation profiling. Some studies emphasise a classification based on traditional morphology alongside sequencing [4], while others base a classification more on epigenetic changes identified on methylation profiling [5–7], which is then supported by morphology, clinical and sequencing correlates.

To some extent, any controversy over technology is artificial, as it can be reframed as a question as to what is meant by a ‘diagnosis’. Does it, for example, describe something inalienable about the biology of the tumour, which might reflect the developmental origin (possibly reflected in the methylation profile), the driving pathway (reflected in the sequencing variants) or the differentiation of the tumour (reflected in the morphology)? Or does it reflect something that directs clinical management, in which case, this might focus on factors that guide prognosis (all modalities), conventional treatment (morphology and to some extent methylation profile) or targeted therapies (dependent on sequencing results)? None of these is intrinsically superior, and the approach needs to be adapted to the setting and clinical question.

The other important factor that frames the discussion is access to resources [8–10]. In well-resourced settings, there should be no reason to choose one modality over another. The costs of multimodal testing are relatively limited compared to those of the neurosurgical operation and subsequent treatment, let alone the costs of a misdiagnosis. For example, in England (https://www.england.nhs.uk/genomics/), all children’s tumours are eligible for methylation profiling, panel sequencing, RNA fusion panels and whole genome sequencing (of the tumour and germline). Faced with this array of data, the pathologist’s role becomes one of balancing differing evidence to form a secure integrated diagnosis. However, these considerations are framed differently in resource-poor countries, where the infrastructure for molecular testing is missing and a more focused tiered approach is necessary [9, 10].

Another consequence of the move to molecular classification is that the historical literature should be treated with caution, as for some tumour types (e.g. low-grade epilepsy-associated tumours [11–14] and PXA [15]), histological classification is not completely reliable, and therefore, historical cohorts are unlikely to represent biologically pure tumour types. Therefore, caution needs to be taken when extrapolating from the conclusions of studies undertaken without full molecular characterisation.

The goal of this paper is not to review the entire spectrum of molecular pathology of low-grade glioma; instead, we have attempted to highlight the key tests likely to offer the most direct way to secure a diagnosis. In doing so, we have omitted descriptions of tests that, at best, provide corroborative evidence but do not definitively solve the diagnosis. This is because, in the current state of molecular knowledge, building a diagnosis on indirect levels of evidence when more specific tests exist is obsolescent, if not wasteful.

In this context, it is worth considering the role of immunohistochemistry. While the mainstay of complex tumour diagnosis for the last three decades, most traditional immunohistochemistry describes patterns of differentiation rather than specific diagnostic data. There are notable exceptions (e.g. mutation-specific antibodies) but these remain a small component of the traditional immunohistochemical repertoire for paediatric low-grade glioma. Therefore, with that exception, the role of traditional immunophenotyping will likely wane as the availability and speed of molecular techniques increase.

### Tumour types

#### Pilocytic astrocytoma

##### Definition

Pilocytic astrocytoma is the archetypal low-grade glioma of childhood. The tumour is often biphasic with a mixture of bipolar cells with thin (hair-like or ‘piloid’) cell processes and stellate cells, along with various mixtures of other characteristic features (e.g. Rosenthal fibres, eosinophilic granular bodies). The tumour is usually driven by a single oncogenic variant in the MAP Kinase pathway (most frequently a *KIAA1549::BRAF* gene fusion) [16]. It is CNS WHO grade 1.

##### Location

Pilocytic astrocytomas can arise almost anywhere in the central nervous system, and rare cases have been described outside the CNS (e.g. in cranial nerves [17]). However, certain sites are typical, e.g. the cerebellum, optic nerves/chiasm, hypothalamus, thalamus, brainstem and spinal cord.

##### Genetic predisposition

Most pilocytic astrocytomas are sporadic. However, they are also the typical CNS tumours arising in neurofibromatosis type 1 (*NF1*). Cases also occur in other germline disorders in which there are variants in the MAP kinase genes (e.g. Noonan’s syndrome (*PTPN11*) and encephalocraniocutaneous lipomatosis (*FGFR1*) [18–20]).

##### Typical histopathology

**Fig. 1:**
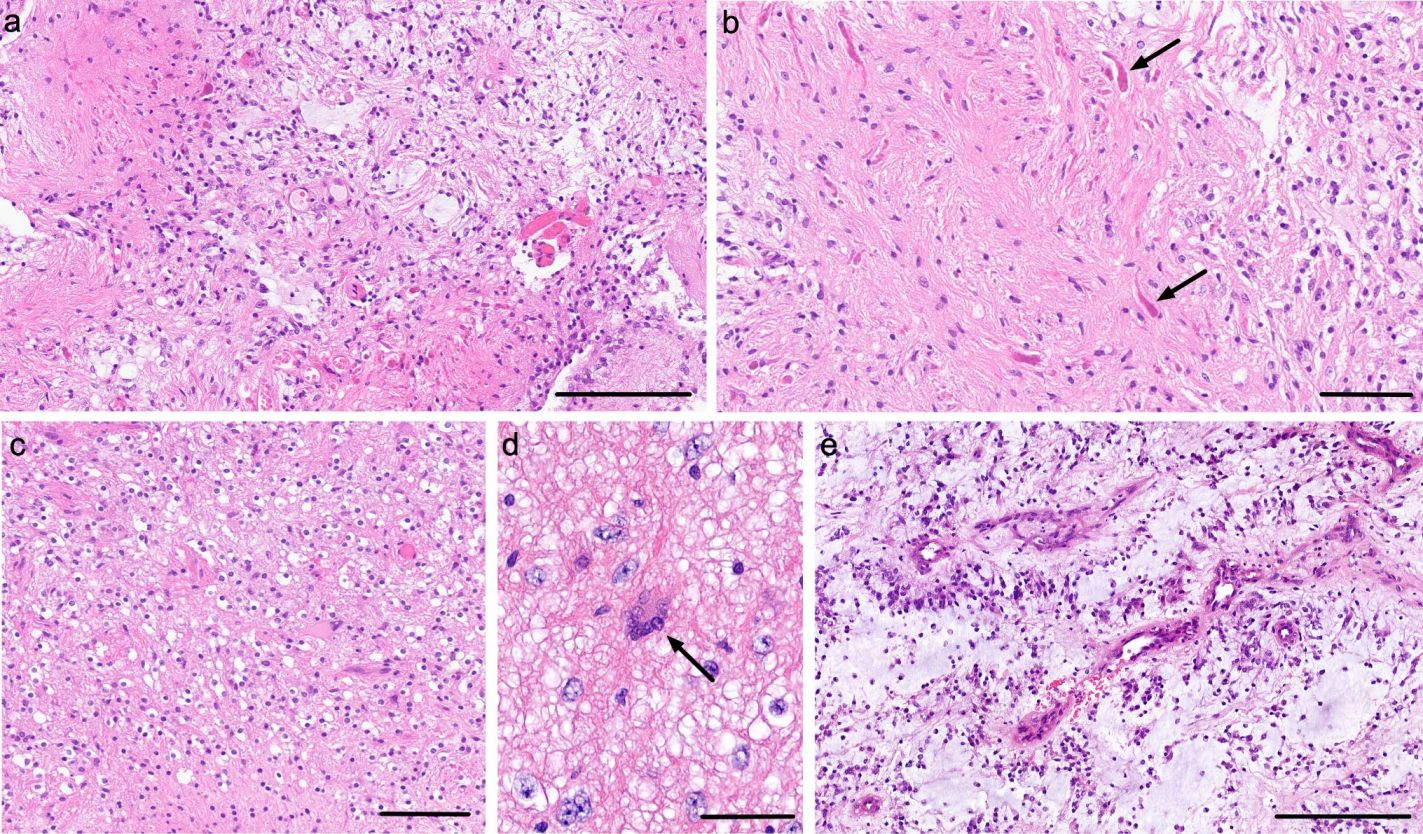
The histopathology of pilocytic astrocytoma. **a** Low-power view showing the biphasic architecture composed of intermixed areas of compact fibrillary stroma containing Rosenthal fibres with looser myxoid areas. **b** Higher power image of the compact fibrillary area. The arrows indicate some of the Rosenthal fibres. **c** A pilocytic astrocytoma with prominent rounded ‘oligodendrocyte’-like areas. **d** A multinucleate cell with a ‘pennies-on-a-plate’ appearance and **e** a pilomyxoid astrocytoma composed of uniform bipolar cells set in a myxoid stroma and exhibiting a perivascular arrangement. Scale bars **a**, **e** 200 μm, **b**, **c** 100 μm, **d** 50 μm. All sections are stained with haematoxylin and eosin

The typical pilocytic astrocytoma is a biphasic tumour composed of intermixed areas of piloid cells set against a compact fibrillary stroma and looser areas of myxoid and microcystic stroma in which the cells may be rounded or stellate (Fig. 1 a, b). The cells typically are monomorphic with inconspicuous mitotic activity.

In many cases, there will be Rosenthal fibres, particularly in the compact areas (Fig. 1 b). Rosenthal fibres are elongated irregular eosinophilic inclusions [21]. They may be seen in a range of low-grade gliomas (particularly pilocytic astrocytoma and ganglioglioma) but can be seen in reactive (piloid) gliosis surrounding slowly expansive lesions (typically craniopharyngioma or haemangioblastoma), in genetic disorders (e.g. Alexander’s disease, giant axonal neuropathy and fucosidosis) and malformations (e.g. focal cortical dysplasia, hemimegalencephaly).

**Fig. 2:**
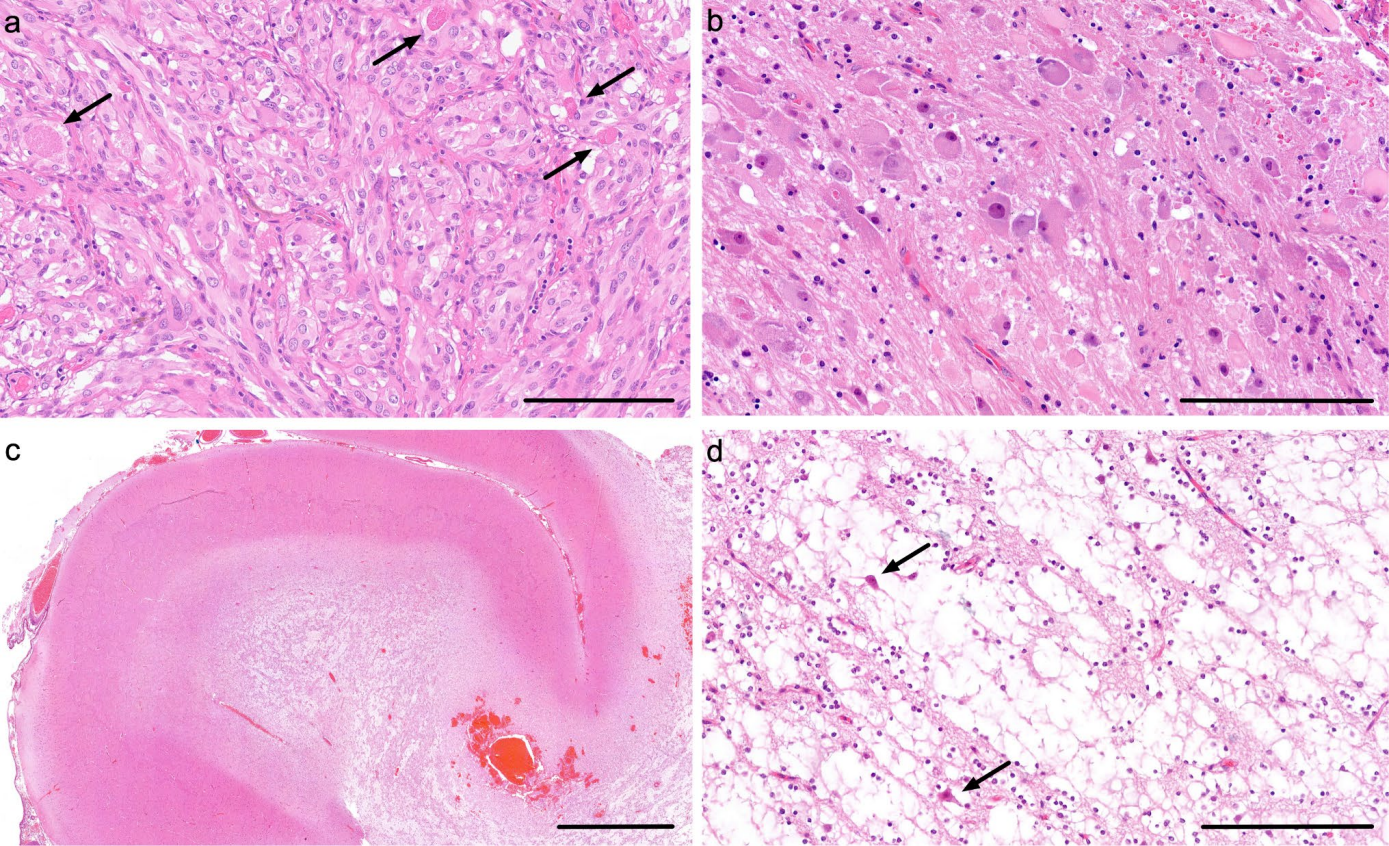
**a** Image of a pleomorphic xanthoastrocytoma in an area composed of pleomorphic plump rounded glial cells. The arrows indicate eosinophilic granular bodies. **b** A ganglioglioma with prominent clusters of abnormal (dysplastic) ganglion cells. **c** Low-power image of a dysembryoplastic neuroepithelial tumour. The image shows a gyrus with an ill-defined nodule in the deep cortex and subcortical white matter. **d** Higher power image of a dysembryoplastic neuroepithelial tumour showing the specific glioneuronal element composed of oligodendrocyte-like cells aligned along fibrillary material separated by myxoid material. The latter contains floating neurons (indicated with arrows). Scale bars **a**, **b**, **c** 200 μm, **c** 3 mm. All sections are stained with haematoxylin and eosin

Eosinophilic granular bodies may also be seen, typically in the looser areas. There are intensely eosinophilic granular inclusions (Fig. 2 a). They can be seen in pilocytic astrocytoma, ganglioglioma and pleomorphic xanthoastrocytoma. They need to be distinguished from other granular or eosinophilic globular structures that may be seen in a range of tumours but lacking the same specificity, and from ovoid bodies, which are more basophilic inclusions, typically seen in areas of previous haemorrhage.

Microvascular proliferation is relatively common in pilocytic astrocytoma and may consist of prominent arcades of proliferating vessels. Necrosis is less frequent but may be seen.

As a common tumour, wide variation in histology is also common, e.g. a typical biphasic pattern may not be seen with only one pattern predominating; there may be a predominance of cells with rounded nuclei and perinuclear haloes (i.e. oligodendrocyte-like cells) (Fig. 1 c); degenerate atypia may be present and can be severe; some cases have a ‘pennies on a plate’ pattern in which rings of nuclei surround eosinophilic cytoplasm (Fig. 1 d); and some show palisading of nuclei. Cases arising in the optic nerve sometimes elicit a profound meningothelial reaction. In addition, rare morphological variants are described (e.g. melanotic pilocytic astrocytoma [22]).

##### Histological subtypes-Pilomyxoid astrocytoma

Pilomyxoid astrocytoma is a morphological variant characterised by uniform bipolar cells set in a myxoid stroma and usually lacking Rosenthal fibres or eosinophilic granular bodies (Fig. 1 e). The tumour cells may have a perivascular arrangement [23, 24]. There are intermediate forms with features of pilomyxoid and typical pilocytic astrocytoma [25].

Pilomyxoid astrocytoma is usually described in young children/infants and arises in the hypothalamus/midline structures. However, the tumour has been described in multiple locations. They have a predisposition to dissemination. The outcome for these tumours is reported to be worse than those of typical pilocytic astrocytoma, but it can be difficult to separate this from issues relating to surgical resection and anatomical location [26].

##### Anaplasia

Some pilocytic astrocytomas show anaplasia (e.g. pleomorphism, mitotic activity and necrosis). In some studies, particularly in adults, such tumours have a worse prognosis [27]. However, how this data applies to children is less clear [28]. Furthermore, the studies may be complicated by the presence of molecularly distinct entities, for example high-grade astrocytoma with piloid features (HGAP) [29]. HGAP is a distinct tumour type which requires molecular testing to distinguish it from pilocytic astrocytoma.

##### Differential diagnosis-Piloid gliosis

In small biopsies, it can be impossible to distinguish pilocytic astrocytobiopsies, it can be impossible to distinguish pilocytic astrocytoma from reactive gliosis, i.e. non-neoplastic tissue surrounding a different primary lesion. In some cases, gliosis can closely mimic features of pilocytic astrocytoma (e.g. showing bipolar cells against a fibrillary stroma containing Rosenthal fibres), and this ‘piloid’ gliosis is particularly common around slowly expansive lesions such as craniopharyngioma and haemangioblastoma.

##### Alexander’s disease

Alexander’s disease is a genetic leukodystrophy characterised by abundant Rosenthal fibres. It may present with a brainstem mass lesion, which on biopsy resembles pilocytic astrocytoma [30]. This presentation is often seen in late-onset forms but may present very early [31]. The presence of Rosenthal-like material in cell bodies (rather than processes) in Alexander’s disease may be the only morphological clue that the biopsy is not from an astrocytoma. Ultimately, genetic testing is required to make the distinction.

##### Diffuse leptomeningeal glioneuronal tumour

Diffuse leptomeningeal glioneuronal tumour (DLGNT) may closely mimic the morphology of pilocytic astrocytoma and clinically, need not be disseminated at presentation. As both tumour types frequently carry *KIAA1549::BRAF* fusions, identification of the genetic driver may be insufficient to distinguish the tumour types. Morphological features that can raise the possibility of DLGNT include rounded oligodendrocyte-like cells with a relative paucity of GFAP. Ultimately, methylation profiling is required to distinguish this entity.

##### High-grade astrocytoma with piloid features

High-grade astrocytoma with piloid features (HGAP) is a distinct tumour type but has morphological overlap with pilocytic astrocytoma and also typically carries a variant driver in the MAPK pathways (often with additional variants in *ATRX* and/or *CDKN2A/B*). Most cases are described in adults, and realistically, this is rarely a diagnostic differential diagnosis of low-grade glioma in children. However, distinction requires methylation profiling.

##### Other low-grade gliomas

If the morphology is not representative or the biopsy is small, it may be difficult to distinguish pilocytic astrocytoma from a range of other low-grade gliomas (including ganglioglioma, PXA and DNET). In such cases, molecular testing is required to refine the diagnosis. As MAPK pathway abnormalities are found in all of these tumours, a methylation profile is required to distinguish these entities when the morphology is not sufficient.

##### Low-grade glioma with H3 mutations

There are rare examples of tumours with either piloid histology associated with a MAPK abnormality and the H3K28M (K27M) which is more typical of a diffuse midline glioma [32, 33]. The classification of these tumours is uncertain, but there is evidence that they may be a distinct tumour type [32].

##### Diagnostic testing

In a typical case, the diagnosis relies only on the morphological appearances of haematoxylin and eosin (H&E)–stained sections. While it is common practice to undertake immunohistochemistry, it rarely adds to the diagnosis.

While morphology is sufficient to secure a diagnosis in most cases, methylation profiling provides strong confirmatory evidence, particularly when the differential diagnosis includes tumour types defined by their methylation profile (e.g. DLGNT, HGAP etc.). Our practice is to undertake methylation profiling on all cases when there is sufficient material to confirm the diagnosis and exclude alternatives. This is of particular value if the clinical presentation is not typical or the progress of the tumour is unusual.

Targeting the MAPK pathway is increasingly used to treat pilocytic astrocytoma, particularly as a primary first-line treatment [34, 35]. Therefore, we routinely undertake testing to identify the driver. There are several possible strategies, but a combination of a panel designed to identify small variants along with an RNA-sequencing-based strategy (e.g. an RNA fusion panel) identifies the driver in most cases. It should be noted that the variant identified is not diagnostic of the tumour type.

##### Pathological prognostic factors

Many histological (mitotic count, necrosis, Ki67 labelling index) and molecular features (e.g. variant type) have been suggested to be prognostic. However, this extensive literature has not led to widely agreed criteria for predicting outcomes. This literature is probably complicated by the lack of full molecular characterisation (e.g. sequencing and methylation profiling) that would allow the exclusion of morphological mimics (e.g. PXA, DLGNT, HGAP). Therefore, it is difficult to prognosticate based on pathological features. A possible exception to this statement is pilomyxoid astrocytoma, which may be associated with a worse outcome.

#### Pleomorphic xanthoastrocytoma (PXA)

##### Definition

The classical definition of pleomorphic xanthoastrocytoma is a morphological one; it is a pleomorphic tumour composed of cells with a mixture of glial and neuronal differentiation, set against a reticulin-rich stroma and showing scattered xanthomatous cells and eosinophilic granular bodies. Such a definition remains at the heart of the WHO classification. However, a molecular definition may better capture this entity’s biology [15]. The defining molecular features include a typical methylation profile, a MAPK pathway abnormality and *CDKN2A/B* loss. Defined in such a way, there is a broader morphological range than the classical histological description. However defined, PXA may be CNS WHO grade 2 or 3.

##### Location

PXA is typically seen as a superficial tumour of the cerebral cortex and the overlying subarachnoid space. However, cases may arise in other locations in the CNS.

##### Genetic predisposition

Most cases are sporadic but rare cases have been described in neurofibromatosis type 1 (*NF1*), Down syndrome (trisomy 21) [36], DiGeorge syndrome (22q11.2 deletion syndrome) [37], Sturge-Weber syndrome (somatic *GNAQ* variants) [38] and familial melanoma-astrocytoma syndrome (*CDKN2A/B* loss) [39].

##### Typical histopathology

The classical pleomorphic xanthoastrocytoma is composed of rounded and spindle-shaped cells with abundant eosinophilic cytoplasm and prominent nuclear pleomorphism (Fig. 2 a). There are frequently eosinophilic granular bodies and variable xanthomatous change. Most textbook descriptions describe extensive pericellular reticulin, but in our experience, particularly outside of the leptomeningeal component, this is an inconstant feature. The tumours typically have a solid growth pattern with a well-defined border with the host brain. Nonetheless, infiltrative areas may be seen. Some of the tumour cells may be overtly neuronal, and this is reflected in the immunophenotype which shows a mixture of glial markers (e.g. GFAP) and neuronal markers. Extensive tumour staining for CD34 is typical and is a feature that has a limited differential diagnosis (principally PXA, ganglioglioma and PLNTY).

Necrosis, microvascular proliferation and raised mitotic activity may occur. A distinction is made between CNS WHO Grade 2 and CNS WHO Grade 3, based on the presence of the mitotic count (≥ 2.5 mitoses/mm^2^).

If a molecular definition is accepted (i.e. a typical methylation class, a MAPK alteration and *CDKN2A/B* loss), the morphological spectrum is much wider and needs to be considered in the context of any moderately cellular astrocytoma [15]. Pleomorphism, xanthomatous change and desmoplasia may be much less prominent. Spindle cells and perivascular arrangements may be more prominent.

##### Differential diagnosis

In the context of low-grade examples, the main differential diagnosis is ganglioglioma and morphological distinction can be difficult. The presence of a *CDK2NA/B* loss and a typical methylation profile favours PXA.

In higher-grade examples, overlap with a wider range of high-grade gliomas should be considered. In particular, a distinction is made in some classifications from epithelioid glioblastoma, but if molecularly defined, the distinction is less clear.

As the morphological spectrum of gliomas with the molecular features of PXA is broad, then PXA should be considered in the differential diagnosis of a wide range of gliomas and glioneuronal tumours.

##### Diagnostic testing

While a morphological diagnosis of PXA is possible in ‘textbook’ cases, the wide range of morphological features in molecularly defined cases and the significant overlap with other gliomas and glioneuronal tumours suggest that molecular confirmation should be the heart of diagnosis. Our practice is to undertake a methylation profile to demonstrate the typical methylation class and the *CDKN2A/B* loss, along with an NGS panel and an RNA fusion panel to identify a MAPK alteration (typically a *BRAF* V600E variant).

##### Pathological prognostic factors

WHO grade predicts outcomes, both in histologically defined cases and in some, but not all, studies of molecularly defined cases [40, 41]. The presence of a *BRAF* abnormality and *CDKN2A/B* loss are definitional rather than prognostic. There is evidence that *TERT* promoter variants may be prognostic [40, 42].

#### Ganglioglioma

##### Definition

Gangliogliomas are low-grade tumours composed of a mixture of dysplastic neurons and neoplastic glial cells. Traditional definitions, including that of the WHO, rely on morphological criteria. However, many studies have shown that morphological criteria are poorly reproducible and that molecular criteria better define subtypes of long-term epilepsy-associated tumours (LEATs), including gangliogliomas. Gangliogliomas are CNS WHO Grade 1.

##### Location

Ganglioglioma can arise throughout the central nervous system but shows a preference for supratentorial regions, with a particular bias towards the temporal lobe that encompasses up to 70% of tumours [43, 44]. The frontal, occipital and parietal lobes may also be affected at a much lower frequency, and ganglioglioma involving the cerebellum, brainstem and spinal cord has been reported in small numbers.

##### Genetic predisposition

The overwhelming majority of gangliogliomas develop sporadically and lack a known underlying predisposition syndrome. However, occasional tumours are identified in patients with neurofibromatosis type 1 [12, 45, 46]. Additionally, there is a suggestion that variants in the tuberous sclerosis genes predispose to ganglioglioma, with several polymorphisms and alterations in *TSC1* and *TSC2* having been identified in these tumours [47, 48].

##### Typical histopathology

Ganglioglioma appears as nodular collections of dysplastic neurons with a distorted, enlarged body and multi-nucleation (Fig. 2b). A neoplastic glial element is found in conjunction with these dysplastic nodules and is typically astrocytic. The proportions and distribution of these core elements can vary significantly, and they may be accompanied by other features. These include calcification, inflammation, Rosenthal fibres and eosinophilic granular bodies.

The immunohistochemical profile of ganglioglioma is not specific, showing a mixture of glial and neuronal markers [49]. CD34 immunopositive cells and processes may be present throughout and in the adjacent cortex. Immunohistochemistry for mutant BRAF (V600E) is frequently positive, with the frequency of BRAF variants in ganglioglioma depending on how the entity is defined (reviewed in [12]).

##### Differential diagnosis-Dysembryoplastic neuroepithelial tumour

Ganglioglioma and dysembryoplastic neuroepithelial tumours (DNET) both affect the temporal lobes and adjacent regions, frequently manifest with refractory seizures and occur predominantly in children. They can be diagnostically challenging to differentiate from one another and are poorly segregated by their histological features [12, 13]. This difficulty is illustrated by poor inter-observer agreement and geographical variability in classifications across surgical series for these tumours, which is not explained by demographic factors and suggests differences in the interpretation of histological criteria for these tumours [49]. Moreover, while the hallmarks of each, dysplastic neurons in ganglioglioma and the specific glioneuronal element in DNET, are specific to each diagnosis, they may not be present in a proportion of cases [13, 50]. Compounding this, other gangliogliomas and DNET-associated histological features appear to show significant overlap between glioneuronal tumours [13].

##### Polymorphous low-grade neuroepithelial tumour of the young (PLNTY)

There is significant overlap both morphologically and genetically between ganglioglioma and PLNTY. Careful molecular assessment is required in the context of the morphological features, but some cases remain ambiguous, likely reflecting some genuine biological uncertainty in separating these entities.

##### Pleomorphic xanthoastrocytoma

Pleomorphic xanthoastrocytoma, being a morphologically diverse glioneuronal tumour, can demonstrate features mirroring ganglioglioma. Moreover, PXA and ganglioglioma share the *BRAF* V600E driver variant and occasional gangliogliomas have been reported carrying *CDKN2A/B* homozygous deletions [46]. Illustrating the potential for misclassification of ganglioglioma, in a recent study of 46 PXAs classified by methylation profiling, 8 had originally been diagnosed histologically as ganglioglioma [41].

##### Cortical dysplasia

Cortical dysplasia and ganglioglioma both present with abnormal neuronal phenotypes and may be difficult to differentiate in cases of ganglioglioma where there is a significant abundance of dysplastic neurons. This is further complicated by the fact that ganglioglioma and other glioneuronal tumours have been reported to associate with cortical dysplasia in the surrounding tissue, termed cortical dysplasia type IIIb [51, 52].

##### Pilocytic astrocytoma

Pilocytic astrocytoma can arise in the temporal lobe and may be associated with epilepsy when this is the case [53]. Additionally, due to variability in the abundance of dysplastic neurons, the tumours may demonstrate the histological appearances of pilocytic astrocytoma. This can be particularly problematic for biopsies and in cases with limited tissue for histological analysis [54].

##### Diagnostic testing

In typical cases, the H&E morphology is sufficient for diagnosis. A range of immunohistochemical testing has been suggested to help in the diagnosis, of which parenchymal CD34 is the most common.

However, as noted above, there is strong evidence that histology alone is poorly predictive of molecular subtypes in LEATs and is poorly reproducible. Therefore, we would advocate methylation profiling and sequencing in all cases for accurate tumour classification.

##### Pathological prognostic factors

The majority of gangliogliomas are indolent tumours with a benign clinical course. Aggressive variants are infrequently encountered and long-term survival rates following resection are positive. In some studies, in addition to histological atypia (pleomorphism, necrosis, elevated mitotic index) and anaplasia, a gemistocytic cell component, lack of protein deposits and focal CD34 staining around tumour cells have been reported as significant predictors of adverse clinical course [55]. Malignant transformation of ganglioglioma is exceptionally rare but small numbers of cases are reported in the literature [43, 56–58]. In these instances, thus far there are no documented biological mechanisms to comprehensively explain why these tumours transform. However, a subset of tumours with malignant transformation to higher grades has been reported to possess an *H3F3A* K27M variant, which was present in the initial tumour in some cases [59, 60].

The status of anaplastic ganglioglioma, which is no longer part of the WHO classification, is uncertain. One study suggested that most cases originally diagnosed as anaplastic ganglioglioma can be reclassified as other tumour types. Those that do not reclassify do not form a distinct tumour subtype by methylation profiling suggesting that there may be no distinct entity of anaplastic ganglioglioma [61].

#### Dysembryoplastic neuroepithelial tumour (DNET)

##### Definition

Dysembryoplastic neuroepithelial tumours (DNET) are nodular cortical low-grade glioneuronal tumours composed of oligodendrocyte-like cells alongside the ‘specific glioneuronal’ element. Traditional definitions, including that of the WHO, rely on morphological criteria. However, many studies have shown that morphological criteria are poorly reproducible and that molecular criteria better define subtypes of LEATs. They are CNS WHO Grade 1.

##### Location

DNET are supratentorial and are very strongly associated with the temporal lobes, in which ~ 80% of tumours are located [50, 62]. Frontal, parietal and occipital lobes are sometimes affected, but with much lower incidence.

##### Genetic predisposition

There are currently no known predisposing factors for the development of DNET, with the overwhelming majority of DNET developing sporadically. Exceptionally rare reports of familial DNET exist with germline *FGFR1* variants [63, 64]. In addition, cases are described in neurofibromatosis (*NF1*) [65] and Noonan syndrome (*PTPN11*) [66].

##### Typical histopathology

Typical dysembryoplastic neuroepithelial tumours demonstrate a nodular intracortical growth pattern and are defined by a hallmark histological appearance termed the ‘specific glioneuronal element’ (Fig. 2 c, d). This is a distinctive columnar arrangement of neuronal fibres and oligodendrocyte-like cells that occurs within a myxoid matrix and may be aligned along vessels [67]. The specific glioneuronal element can contain normal-appearing neurons that lack apparent connections to their adjacent environment, described as ‘floating neurons’. It is unclear whether these neurons represent an active pathological element or cortical neurons entrapped by a purely glial tumour [68]. The specific glioneuronal element is sometimes accompanied by glial nodules with abnormal astrocytic or oligodendrocytic elements.

Three histological subtypes of DNET have been proposed based on the presence of these hallmark features: complex, simple and non-specific or diffuse. The complex form contains both the specific glioneuronal element and glial nodules [68]. The simple form is composed only of the specific glioneuronal element without glial nodules. There is significant disagreement about the acceptance of the non-specific or diffuse form as a legitimate histological subtype [49]. This proposed form is composed of only glial nodules and the specific glioneuronal element is absent, with an absence of other distinguishing histological features [69].

In addition to the specific glioneuronal element and glial nodules described previously, other features may be present. These include calcification, white matter rarefaction, Rosenthal fibres and eosinophilic granular bodies [50, 62]. These features are markedly variable in their reported frequency and, as previously noted for ganglioglioma, are of limited use in distinguishing DNET from other tumours.

##### Differential diagnosis-Ganglioglioma

As previously described, ganglioglioma and DNET can present a diagnostic challenge due to overlapping presentation, demographics, location and histology. This is particularly problematic in cases where the hallmark features of each, dysplastic neurons in ganglioglioma and a specific glioneuronal element in DNET, are absent. We advocate a molecular distinction between differing types of LEAT.

##### Polymorphous low-grade neuroepithelial tumour of the young (PLNTY)

Polymorphous low-grade neuroepithelial tumour of the young (PLNTY) by definition will have oligodendroglial-like cells and therefore enters the differential diagnosis of DNET.

##### Pilocytic astrocytoma

Pilocytic astrocytoma, specifically those occurring in the cerebral hemispheres, is a potential differential diagnosis for DNET. Both primarily affect young patients and they share several possible histological features including microcystic change, eosinophilic granular bodies, Rosenthal fibres, involvement of the leptomeninges and vascular proliferation [62].

##### Oligodendroglioma

Oligodendrogliomas frequently involve the cortex and are associated with seizures. They are much more common in adults than children, and thus only represent a differential diagnosis for DNET in older patients. Oligodendrogliomas are defined by the presence of an IDH mutation and 1p19q co-deletion, and therefore molecular testing can resolve any confusion.

##### Diagnostic testing

In typical cases, the H&E morphology is sufficient for diagnosis. However, as noted above, there is strong evidence that histology alone is poorly predictive of molecular subtypes in LEATs and is poorly reproducible. Therefore, we would advocate methylation profiling and sequencing in all cases for accurate tumour classification.

##### Pathological prognostic factors

DNETs are typically benign. In the original series of 39, no patients succumbed to tumour-related factors and recurrence was not evident over a mean 9-year follow-up period [67]. This trend was repeated across subsequent large cohorts [50, 62, 69]. In rare, isolated cases, aggressive tumours have been reported that appear to experience malignant transformation. A review on DNET malignant transformation conducted by Moazzam et al*.* in 2014 suggested only 10 instances of progression to a higher grade in the literature [70]. The majority of these cases were complex DNET and common factors across the collected cohort were extratemporal location and subtotal resection. Takita et al*.* expanded upon this in 2022, identifying a total of 14 cases of DNET with malignant transformation in the literature, recapitulating extratemporal location as a common factor [71].

#### Diffuse leptomeningeal glioneuronal tumour

##### Definition

Diffuse leptomeningeal glioneuronal tumour (DLGNT) is a glioneuronal tumour comprising oligodendrocyte-like tumour cells, often with widespread or diffuse involvement of the leptomeninges. The molecular features are characterised by a typical methylation profile, a MAPK pathway alteration (typically *KIAA1549::BRAF* fusion) and chromosome 1p deletion [72]. It is not currently graded in the WHO classification, although the tumour aligns most closely to CNS WHO grade 2 or 3.

##### Location

They most frequently involve the leptomeninges of the spinal cord, followed by the posterior fossa or brain stem, and less commonly the cerebral hemispheres. They more rarely arise within the parenchyma alone.

##### Predisposition

There is no established genetic predisposition but individual cases have been described in patients with 5p deletion, type 1 Chiari malformation and factor V Leiden mutation [73].

##### Typical histopathology

**Fig. 3:**
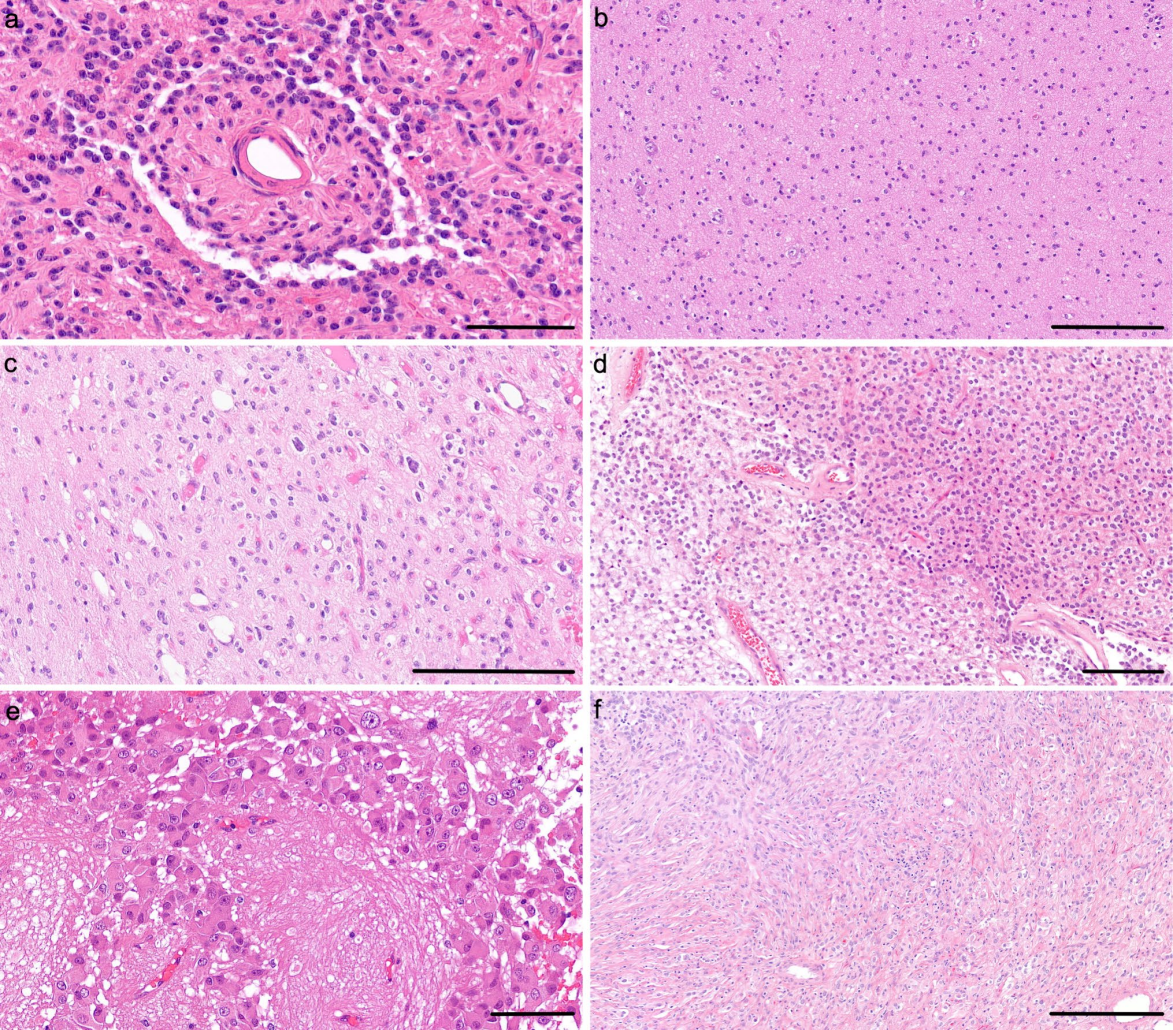
**a** Image of an angiocentric glioma showing cells surrounding a blood vessel. **b** A low-power image of a diffuse astrocytoma, *MYB/MYBL1*-altered showing diffuse sheets of unremarkable glial cells set against a fibrillary stroma. **c** Polymorphous low-grade neuroepithelial tumour of the young (PLNTY) showing rounded oligodendrocyte-like cells with mild pleomorphism. **d** A diffuse leptomeningeal glioneuronal tumour showing diffuse sheets of rounded oligodendrocyte-like cells. **e** Subependymal giant cell astrocytoma composed of plump cells with eosinophilic cytoplasm. There is a prominent perivascular arrangement in this case. **f** Desmoplastic infantile ganglioglioma showing the desmoplastic component. Scale bars **a** 70 μm, **b**, **c**, **d** 200 μm, **e** 90 μm, **f** 300 μm. All sections are stained with haematoxylin and eosin

The tumours usually have low to moderate cellularity and mainly comprise oligodendroglia-like tumour cells with uniform round nuclei and perinuclear haloes, arranged in nests or sheets, with myxoid and desmoplastic changes (Fig. 3 d). The tumour cells are positive for OLIG2 and synaptophysin but may lack GFAP. Most but not all tumours lack high-grade or anaplastic features such as brisk mitotic activity, significant nuclear pleomorphism, necrosis or microvascular proliferation.

##### Histological variation

Rarely, they can exhibit anaplastic features [74] and a small subset of tumours may contain overt neuronal differentiation.

##### Differential diagnosis

The common histological mimics to consider are pilocytic astrocytoma and dysembryoplastic neuroepithelial tumour in children, and oligodendroglioma in adults.

##### Diagnostic testing

These tumours are defined by their methylation profile which should be undertaken to secure the diagnosis [72]. In addition, they are characterised by chromosome 1p deletion and a MAPK pathway alteration, commonly a *KIAA1549::BRAF* fusion.

##### Pathological prognostic factors

A recent systematic review has reported Ki-67 of ≥ 7% as a significant predictor of poor survival [75]. Molecularly, tumours of methylation subclass 2 (DLGNT-MC-2) are known to have more aggressive behaviour than subclass 1 (DLGNT-MC-1) [72]. A gain of 1q has also been shown to be an adverse prognostic factor [76]. Integration of pathological and molecular features with clinical and imaging findings would be of the essence for this tumour entity [77].

#### MYB and MYBL1-altered tumours

These are a group of diffuse gliomas in which there are structural variants in the *MYB* or *MYBL1* genes. Two morphological subtypes are recognised: angiocentric glioma and diffuse astrocytoma, *MYB*- or *MYBL1*-altered.

#### Angiocentric glioma

##### Definition

Angiocentric glioma is a low-grade tumour composed of uniform bipolar cells showing, at least focally, an angiocentric pattern. Most cases carry a *MYB* gene alteration, most commonly a *MYB::QKI* fusion [78]. The tumours are CNS WHO grade 1.

##### Location

This tumour typically arises in the cerebral cortex, but other sites (such as the brainstem) are well recognised.

##### Genetic predisposition

No genetic predisposition has been identified, but rare cases are described in neurofibromatosis type 1 and Koolen-de Vries syndrome (17q21.31 microdeletion syndrome) [79, 80].

##### Typical histopathology

The tumour is composed of cytologically bland, uniform bipolar spindle cells with slender oval nuclei (Fig. 3 a). The tumour cells can show variable architectural features including solid parts, diffuse infiltrative parts and subpial accumulation, but the diagnostically distinctive pattern is perivascular, with tumour cells often arranged around the blood vessels in a radial or rosette-like pattern, exhibiting ependymal differentiation. The tumour lacks high-grade features (there is little mitotic activity, and no necrosis or microvascular proliferation in typical cases). There may be scattered neurons between the tumour cells but these are not dysplastic. The tumour cells will express markers of glial differentiation (e.g. GFAP). They show ependymal differentiation on immunophenotype (e.g. with EMA-dot positivity/lumens and lack of OLIG2 expression) and electron microscopy.

##### Histological variation

There are no recognised histological subtypes described, although some tumours may have epithelioid changes. Very rare tumours with anaplastic histology [81] have been reported but are of uncertain clinical significance.

##### Differential diagnosis

Typical cases raise little diagnostic challenge, but the differential diagnosis includes other low-grade gliomas and in some cases, other tumours showing ependymal differentiation, e.g. ependymoma.

##### Diagnostic testing

In a typical case, the diagnosis can be made with confidence on the H&E-based morphology alone, but confirmation by the demonstration of a *MYB* alteration, most frequently with fusion of *MYB::QKI* genes [78, 82], and a typical methylation profile is recommended.

##### Pathological prognostic factors

These tumours are known to have a favourable prognosis with gross total resection, without the need for radiation or chemotherapy [83]. There are no known prognostic or predictive factors at this stage.

#### Diffuse astrocytoma, MYB- or MYBL1-altered

##### Definition

This is a low-grade diffuse glioma with an alteration in the *MYB* or *MYBL1* genes. They are CNS WHO grade 1.

##### Location

These are typically tumours of the cerebral cortex (presenting with epilepsy) but brainstem cases occur.

##### Genetic predisposition

There is no known genetic predisposition.

##### Typical histopathology

The tumours show a diffuse pattern composed of relatively unremarkable glial cells set against a loose fibrillary stroma replacing the cerebral cortex and superficial white matter [84] (Fig. 3 b). There may be remaining neurons within the tumour, but these are reduced in number. The tumour cells usually lack pleomorphism and mitotic activity, and there is no necrosis or microvascular proliferation. Indeed, the morphological features may be so subtle as to not suggest a tumour at all. In contrast, some cases show mild pleomorphism with increased mitotic activity.

##### Differential diagnosis

In cases lacking any anaplasia, the differential diagnosis is often with reactive (non-neoplastic) gliosis, and it is the recognition of the possibility of a diffuse astrocytoma that makes the diagnosis possible. The other differential diagnoses are other subtypes of diffuse glioma.

##### Diagnostic testing

The typical morphological features will raise a strong histological suspicion of the diagnosis, but molecular confirmation of the *MYB/MYBL1* structural variant and typical methylation profile secure the diagnosis. It should be noted that not all *MYB/MYBL1* structural alterations can readily be detected by typical diagnostic techniques, and their absence in the presence of a typical methylation profile should not exclude the diagnosis.

##### Pathological prognostic factors

Most patients have an excellent outcome and well-described prognostic features based on pathology have not been identified [84]. Some cases do show higher-grade morphological features, but the prognostic significance of these findings is uncertain.

#### Desmoplastic infantile astrocytoma/ganglioglioma (DIA/DIG)

##### Definition

Desmoplastic infantile astrocytoma/ganglioglioma is a tumour of the cerebral hemispheres arising in infancy characterised by the presence of a mixture of a desmoplastic spindle tumour, a glial or glioneuronal component and a poorly differentiated small cell component [85]. They are CNS WHO Grade 1.

##### Location

The tumours typically arise as large solid/cystic superficial supratentorial lesions affecting the cortex and leptomeninges.

##### Genetic predisposition

There is no known genetic predisposition.

##### Typical histology

The tumour has several different components [85, 86]. Many tumours show a predominant spindle cell component arranged in a storiform pattern with a dense desmoplasia on reticulin staining (Fig. 3 f). There is minimal pleomorphism or mitotic activity in this component. There may be an admixed overtly glial or glioneuronal component set against CNS-type stroma. Finally, there may be a small cell component composed of poorly differentiated cells, which may show anaplasia and mitotic activity.

##### Differential diagnosis

The main morphological differential diagnoses include conventional ganglioglioma, pleomorphic xanthoastrocytoma and infant-type hemispheric glioma. The small cell component may raise the possibility of an embryonal tumour or other high-grade tumour.

##### Diagnostic testing

In typical cases, the morphology may be sufficiently typical for diagnosis, but molecular testing including sequencing (for a MAPK alteration) and methylation profiling secures the diagnosis and excludes alternatives.

##### Pathological prognostic factors

Most cases have an excellent prognosis. In particular, the presence of the poorly differentiated small cell component does not worsen the prognosis. A few patients may have more overt anaplastic features and some cases recur, but histological features that predict a worse outcome have not been agreed upon.

#### Subependymal giant cell astrocytoma (SEGA)

##### Definition

Subependymal giant cell astrocytomas are benign, circumscribed slow-growing gliomas arising from the ventricular walls associated with tuberous sclerosis complex (TSC) and therefore driven by variants in *TSC1* or *TSC2*. It is classified as a CNS WHO grade 1 tumour.

##### Location

It typically appears in the first two decades of life in the lateral ventricle near the foramen of Monro, but rare cases have been described at other sites.

##### Genetic predisposition

SEGA is characteristically seen in patients with TSC, an autosomal dominant phacomatosis due to inactivating variants in *TSC1* or *TSC2*, genes that encode for inhibitors of the mammalian target of rapamycin (mTOR). It affects up to 27.5% of TSC patients [87], constituting one of the major diagnostic criteria for this disease [88]. Rare cases are described outside of TSC.

##### Typical histopathology

SEGA are moderately cellular tumours composed of mixed glial and neuronal cells. The characteristic cytological appearances include large polygonal cells with abundant glassy cytoplasm and well-defined cytoplasmic membranes, mixed with gemistocyte-like cells and smaller spindled cells (Fig. 3 e). Ganglion-like cells may be seen. There is usually marked nuclear pleomorphism and multi-nucleation. The tumour can be arranged in sheets, fascicles and nests occasionally delineated by fibrous septa. Perivascular pseudo-rosettes are a prominent feature in some tumours. A chronic inflammatory infiltrate composed of lymphocytes and mast cells is observed. Calcification is frequently observed. Most cases lack increased mitotic activity, microvascular proliferation or necrosis, but their presence does not imply a change in diagnosis or grade per se.

The tumour immunoprofile shows a mixture of glial and neuronal markers [89–91]. Nuclear expression of thyroid transcription factor-1 (TTF-1) is typical in SEGAs [92].

##### Differential diagnosis

In the correct clinical context, the morphological features are typical, and there is no significant differential diagnosis. However, the presence of large epithelioid cells with significant pleomorphism in addition to possible atypical features (such as mitoses, necrosis or microvascular proliferation) can raise the differential of high-grade gliomas, pleomorphic xanthoastrocytoma or ganglioglioma (e.g. see [93]). In cases with perivascular pseudo-rosettes, ependymoma may be considered.

A glioma histologically resembling SEGA has been described in patients with neurofibromatosis type 1 (*NF1*) [94].

##### Diagnostic testing

Most SEGAs can be identified with confidence based on the H&E appearance. Immunohistochemistry adds little diagnostic information in most cases. SEGAs have a distinct methylation profile and can be used in tumours with equivocal histology [7, 89, 95]. Identification of germline pathogenic variants in *TSC1* or *TSC2* genes suffice for the diagnosis of TSC in patients without a confirmed clinical diagnosis. In rare instances, SEGA can occur in patients without germline TSC variants, although somatic TSC variants have been described in these tumours [96].

##### Pathological prognostic factors

SEGAs are associated with a good prognosis and histopathological features are not used to stratify outcomes.

#### RGNT

##### Definition

Rosette-forming glioneuronal tumour (RGNT) has two components, a neurocytic one with rosettes/pseudo-rosettes and a glial component resembling a pilocytic astrocytoma. It is classified as a CNS WHO grade 1 tumour.

##### Location

RGNT is typically a tumour of the fourth ventricle with local extension to adjacent structures, such as the brainstem and cerebellar vermis. Other locations include the quadrigeminal cistern, cerebellar hemispheres, pineal gland, third ventricle, thalamus, suprasellar region, optic chiasm, spinal cord, frontal lobe and temporal lobe [97–104]. CSF dissemination and drop metastasis have been described, but are not common [100, 102, 105–107].

##### Genetic predisposition

RGNTs are usually sporadic but have been described in patients with neurofibromatosis type 1 and Noonan syndrome [108–110].

##### Typical histopathology

The tumour has a low to moderate cellularity and exhibits a dual histological morphology, with neurocytic and glial components. The former is composed of monomorphic neurocytic cells arranged in pseudo-rosettes with eosinophilic neuropil cores and/or perivascular pseudo-rosettes, which are embedded in a microcystic or mucinous stroma. Cytologically, the neurocytes have rounded nuclei, fine granular chromatin, inconspicuous nucleoli and scant cytoplasm with delicate processes. The glial component can resemble a pilocytic astrocytoma and may comprise much, if not most, of the specimen. It is characterised by stellate or spindled astrocytic cells with oval nuclei; its fibrillary processes form a loose to compact stroma. Some tumours show oligodendroglioma-like areas, with glial cells displaying rounded nuclei and perinuclear haloes, set in a microcystic stroma. Eosinophilic granular bodies and Rosenthal fibres are occasionally present. Ganglion cells can be seen. Vascular changes include vessels with hyalinised walls, glomeruloid proliferation and thrombosis [111, 112]. Mitoses are usually inconspicuous and the Ki-67 proliferation index is low.

The glial and neurocytic components show distinct immunophenotypes that reflect glial and neuronal differentiation respectively.

##### Differential diagnosis

The tumour has overlapping histological features with other low-grade neuroepithelial tumours, particularly those with *FGFR1* alterations, such as pilocytic astrocytoma, extraventricular neurocytoma and dysembryoplastic neuroepithelial tumours [103, 113].

##### Diagnostic testing

In typical cases, the morphology is diagnostic. However, small samples or those in which all the features have not been sampled may be problematic. RGNT has a distinct DNA methylation signature [7]. RGNTs harbour *FGFR1* kinase domain hotspot missense variants (N546K, D652G, K656E), with co-occurrence of mutually exclusive activating variants in *PIK3CA* or inactivating variant in *PIK3R1* [103, 113–118]. There may be additional *NF1* or *PTPN11* variants [113].

##### Pathological prognostic factors

RGNTs have a predominantly indolent course. Rare cases of anaplastic transformation, drop metastasis or recurrence have been described in the literature [102, 105, 107, 119–121]. However, histopathological features that predict a worse outcome have not been identified.

#### Polymorphous low-grade neuroepithelial tumour of the young (PLNTY)

##### Definition

Polymorphous low-grade neuroepithelial tumour of the young (PLNTY) is a morphologically variable tumour associated with seizures. The defining features are moot, as the original definition relied on their methylation profile, and while subsequent studies have suggested that certain molecular features assist in defining the tumour more precisely, most papers and indeed the WHO classification rely predominantly on histological features along with a MAPK pathway abnormality. The histological features used to define them include an oligodendroglioma-like component, infiltrative growth patterns and CD34 immunoreactivity. They are CNS WHO Grade 1.

##### Location

PLNTY most frequently arise in the temporal lobe, representing ~ 70% of tumours in the literature thus far [122]. They can also be found at much lower rates affecting the occipital, frontal and parietal lobes.

##### Genetic predisposition

Thus far, no genetic predisposition syndrome or predisposing factors have been associated with the development of PLNTY. A rare case has been described associated with a germline *ATM* variant [123].

##### Typical histopathology

The typical histology of PLNTY is highly variable, demonstrating striking intra- and intertumoural heterogeneity. However, in the original descriptive cohort, all tumours displayed an infiltrative growth pattern and oligodendroglioma-like components with minimal or absent mitoses [124] (Fig. 3 c). The oligodendroglial element ranged from uniform small, rounded cells with a clear halo to components with considerable variation in nuclear size and shape. Huse et al*.* also described oval and spindled nuclear contouring as well as nuclear membrane wrinkling, grooving, and intranuclear pseudo-inclusions in some cases. A proportion of tumours may also demonstrate pseudo-rosetting of neoplastic cells around blood vessels. In addition to this oligodendroglioma-like component, almost all tumours in the original cohort possessed an astrocytic element which included fibrillary, spindled and pleomorphic populations of varying density. Calcification is also common throughout the tumour in most cases. Gemistocytic elements, Rosenthal fibres, eosinophilic granular bodies, myxoid micro-cysts, dysplastic neurons, neurocytic/ependymal rosettes, microvascular proliferation and necrosis are all noted as being absent in PLNTY. These histological trends have followed through in subsequent PLNTY cohorts [125, 126].

The immunohistochemical profile of PLNTY is dominated by widespread expression of CD34 in tumour cells, though in some cases staining may be patchy or focal. In addition, CD34-positive neural elements are present in the adjacent cortex [124]. GFAP and OLIG2 expression is widespread, if sometimes patchy or focal for the former.

##### Differential diagnosis

In children, the primary differential diagnoses of PLNTY are other low-grade glioma and glioneuronal tumours. Here, the main distinction for PLNTY at the histological level is widespread CD34 expression, which is generally absent in other low-grade diffuse gliomas [127–129]. They can also be distinguished by their methylation profile, which is similar to but distinct from other low-grade neuroepithelial tumours, though there may be some overlap with ganglioglioma [124].

##### Diagnostic testing

While the WHO classification advocates a predominantly morphological diagnosis, there remain significant grey areas over the diagnosis of PLNTY compared to other glioneuronal tumours, particularly ganglioglioma. It is, therefore in our opinion, prudent to not only confirm the presence of a MAPK pathway abnormality but to undertake methylation profiling to help distinguish the tumour type. There is evidence that the presence of an *FGFR2* fusion may help secure the diagnosis [130].

##### Pathological prognostic factors

Survival figures for the limited number of PLNTY in the literature are good, and recurrence is rare, in line with other low-grade epilepsy-associated tumours. In the original series, a single patient who underwent gross total resection demonstrated potential evidence on radiology of a new area of abnormality [124]. In another cohort, one patient demonstrated progressive recurrence 60 months after total resection [123]. Thus far, only a single tumour with malignant transformation to a higher grade has been reported [131]. This patient developed a partially solid and cystic lesion 17 months after gross total resection for PLNTY, with histological analysis of the recurrent tumour demonstrating a lack of CD34 expression and foci of high-grade features. In addition to an *FGFR3::TACC3* fusion, this tumour possessed somatic alterations in *TP53*, *ATRX*, *PTEN*, *TEK*, and *RB1*.

#### Diffuse low-grade glioma, MAPK pathway-altered

This tumour class was introduced in the 2021 WHO classification. There are different approaches to diagnosing diffuse low-grade gliomas in children, and it was introduced to capture a spectrum of diffuse gliomas without other distinctive features in which there was a single variant in a gene in the MAPK pathway (typically *BRAF* or *FGFR1* showing respectively morphologically astrocytic or oligodendroglial features). The concept builds on those previously outlined in cIMPACT-NOW update 4 in which a matrix or layered diagnosis can be offered in diffuse low-grade paediatric gliomas where morphological features are matched with the variant [132]. This approach is supported by some of the published prognostic classifications [133].

In contrast, it should be noted that this tumour class has not been shown to have a distinctive methylation profile; it seems probable that tumours with these morphological and molecular features span a range of methylation classes. Therefore, it is unclear whether this class of tumours represents a true distinct entity, a histo-molecular pattern of a range of tumours or a mixture of both. From a practical perspective, our approach when considering this diagnosis is to undertake extensive molecular testing, including methylation profiling, panel sequencing for small variants and fusions and where possible whole genome sequencing, and to only use this diagnosis if a more specific diagnosis cannot be achieved, which in our experience is rare.

## Conclusion

Detailed pathological diagnosis provides the optimal basis for management decisions for both conventional treatments and targeted therapies. While diagnostic classification based on morphology remains the foundation of pathological diagnosis, and in some cases may be sufficient, the redefinition or definition of many tumour types by molecular classification has shifted the morphological spectrum seen in those tumour types, necessitating greater use of molecular testing to avoid misdiagnosis. Furthermore, targeted treatment depends on the accurate identification of the driver variant.

In our practice, we undertake a combination of traditional morphology, methylation profiling and sequencing in most cases. This means that diagnosis is supported not only by the subjectivity of expert opinion (i.e. morphology) but at least one if not two objective tests (e.g. methylation classification and variant identification) reducing the likelihood of a diagnostic error. This also has the advantage of improving the accuracy of the expert opinion of the morphology as this is informed by regular feedback (discussed in [134]).

It is likely as treatment regimens for low-grade gliomas shift towards long-term targeted treatment, the pathological diagnosis of low-grade gliomas will shift its emphasis to identifying factors that predict response, resistance and rebound following treatment and to understanding the biology of long-term disease. This direction of travel will emphasise the need for pathological assessment of late-stage disease.

## Data Availability

No new data was created or analyzed during this study. Data sharing does not apply to this article.
